# Morphogenesis in Fungal Pathogenicity: Shape, Size, and Surface

**DOI:** 10.1371/journal.ppat.1003027

**Published:** 2012-12-06

**Authors:** Linqi Wang, Xiaorong Lin

**Affiliations:** Department of Biology, Texas A&M University, College Station, Texas, United States of America; Duke University Medical Center, United States of America

## Introduction

Morphological changes are required for eukaryotic pathogens to cause disease. However, it is only now becoming clear how such transitions are linked to virulence in human pathogenic fungi. Changing cell size and shape are strategies employed by many of these fungi to survive in the environment and serendipitously also within the host. Conserved signaling pathways regulate morphogenic differentiation in response to environmental and host physiological stimuli. The alterations in cell-surface composition during morphogenesis, in addition to cell size and shape, further link virulence with morphogenesis.

## Morphotype Transition Is Associated with Fungal Virulence

Changes in morphology are required by diverse microbes to be successful pathogens, and the well-known examples include bacterial pathogen *Chlamydia trachomatis* and protozoan parasite *Plasmodium falciparum*. Morphotype transitions are also required for fungi to cause disease in plants. For example, the rice blast ascomycete *Magnaporthe oryzae* undergoes a series of morphotype changes (conidia→appressoria→penetration peg→invasive hyphae) for infection. The corn smut basidiomycete *Ustilago maydis* grows as a yeast in vitro and infects plants only in the hyphal form after mating.

**Figure 1:**
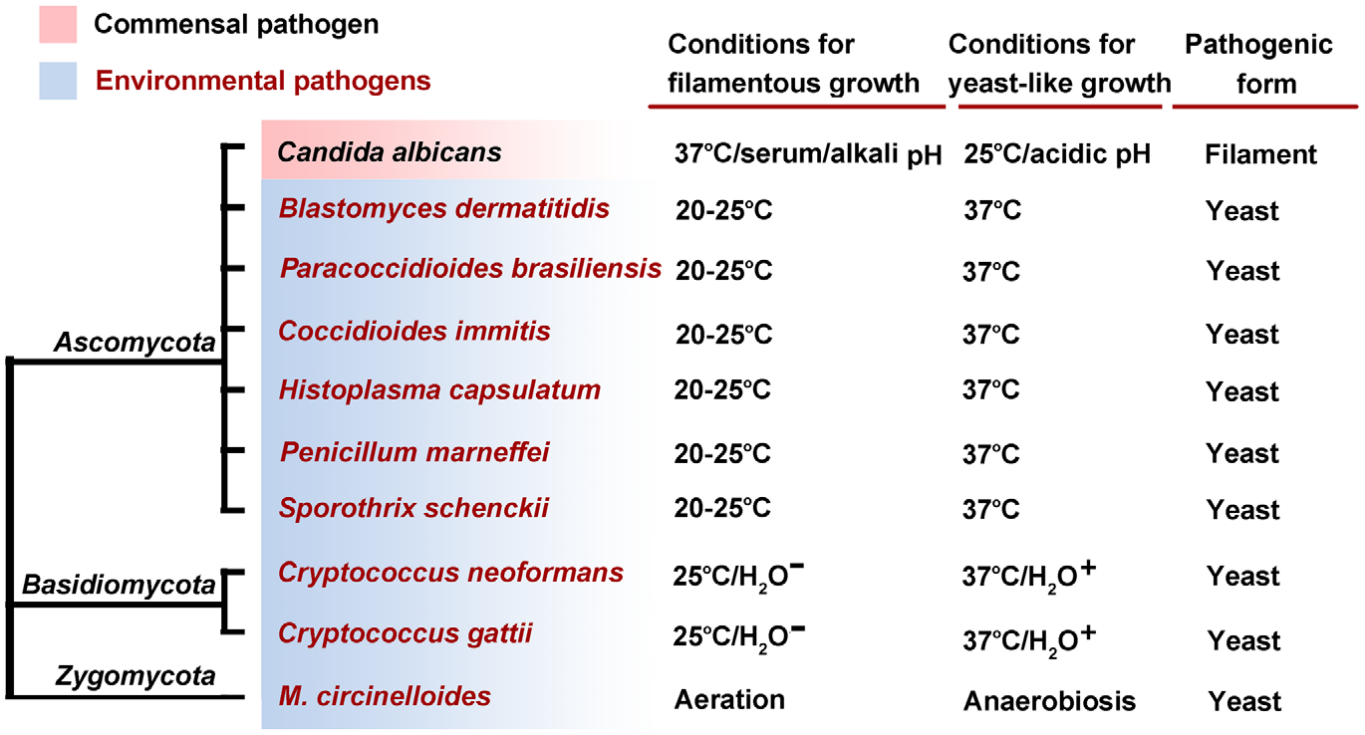
Fungal pathogens from different phyla exhibit a dimorphic lifestyle. *Candida albicans* is a commensal or opportunistic pathogen, distinguishing it from the other species that are acquired from environmental exposure. The factors regulating dimorphism are provided: H_2_O^−^ dehydration; H_2_O^+^ aqueous environment.

The majority of fungi grow either filamentously by apical extension or unicellularly by budding or fission. A few human pathogenic fungi switch between these two growth forms, and these are called dimorphic fungi. Here, “morphotype transition” or “dimorphism” refers to the ability to switch between two or more different growth forms. It has been established in the “classic” dimorphic pathogens (listed in the clade of Ascomycota in Figure 1) that proper morphotype transition is required for virulence: blocking transitions by chemicals or genetic mutations attenuates or abolishes their ability to cause diseases [1]–[6].

Morphotype-associated pathogenicity is likely more widespread than just those species traditionally classified as dimorphic. For instance, *Cryptococcus neoformans*, a basidiomycetous pathogen, has both a yeast and a filamentous form. Early studies on natural *Cryptococcus* isolates implicated an inverse relationship between filamentation and virulence (see [7] and references therein). A molecular link between morphogenesis and virulence in this fungus was obtained recently through the characterization of the transcription factor Znf2. Overexpression of the *ZNF2* gene confers filamentation and abolishes the ability of this fungus to cause disease, while the deletion of the *ZNF2* gene locks cells in the yeast form and enhances virulence [8], [9]. The existence of morphotype-associated pathogenicity is also observed in some zygomycetes [10] and in dermatophytes [11]. Thus, it appears that pathogens of different major phyla in the fungal kingdom have adopted dimorphism as a common pathogenic strategy. The underlying question is what advantage this provides to these species.

## Changes in Cell Size Impact Fungal Pathogenicity

Cell size affects multiple steps during infection and disease progression. As infectious propagules, spores of small sizes are ideal for dispersal and entry into host lungs. For instance, *Cryptococcus* spores (1–2 µm in diameter) are easily lodged into the lower respiratory tract and phagocytosed by lung macrophages [12], [13]. Spores then germinate into encapsulated yeasts of varied sizes. These yeast cells can withstand the antimicrobial activities mediated by macrophages, proliferate intracellularly, and escape from the host cell. Mycelia of *Histoplasma capsulatum* produce two types of conidia, macroconidia (8–16 µm), and microconidia (2–5 µm). The microconidia enter the alveolar spaces and germinate into budding yeasts that can persist intracellularly in phagocytotic cells, a critical step for *Histoplasma* infection [14]. However, for fungal pathogens that typically infect the upper respiratory tract, gastrointestinal tract, or subcutaneous tissues, cell size has a different impact on virulence. The zygomycete *Mucor circinelloides* f. *lusitanicus* is a heterothallic fungus with (+) or (−) mating types. Asexual spores produced by (−) strains are larger and can germinate inside macrophages and lyse host cells. Accordingly, the spores produced by (−) strains are more virulent [10]. A key observation is that allowing small spores to germinate and develop into large cells in vitro, prior to the infection, increases the virulence of the inoculum [10].

Heterogeneity in cell size is a bet-hedging strategy that poises a population of cells of identical genotype for different contingencies in the host. For instance, a subset of *Cryptococcus* yeast cells enlarge from the typical 3–5 µm to up to 100 µm in diameter during later stages of lung infection [15], [16]. These giant cryptococcal cells can evade phagocytosis and better tolerate oxidative and nitrosative stresses. A mutant defective in producing these large cells has attenuated virulence [17]. Similarly, *Paracoccidioides brasiliensis* wild-type populations show varied cell size, and mutant cells homogenously reduced in size are more susceptible to phagocytosis by macrophages and are unable to cause disease [18].

## Changing Cell Shape or Size May Have Evolved as a Response to Environmental Stresses

The majority of the human fungal pathogens are environmental saprophytes (Figure 1), and their ability to survive in a mammalian host is a consequence of selective pressures posed by their environment. Changing cell shape or size is likely an adaptive trait that is also advantageous when these pathogens encounter the host. Accordingly, signaling pathways that play important roles in coordinating morphological changes and virulence (see the next section) are typically involved in sensing environmental stresses.

Of these stresses, high body temperature presents a major barrier for many fungal species to infect mammals. This barrier may explain the rarity of fungal diseases in humans, in contrast to the prevalence of fungal diseases in plants, insects, amphibians, or even bats during hibernation [19]. Interestingly, elevated temperatures are the primary signal directing dimorphic fungi to switch from the saprotrophic filamentous phase to the pathogenic yeast phase (Figure 1).

## Conserved Signaling Pathways Orchestrate both Morphogenesis and Virulence

Ancient signaling pathways that respond to environmental cues control fungal morphogenesis and virulence. These include the cAMP/PKA pathway, the PKC cell wall integrity pathway, the pheromone sensing pathway, the Ca^2+^-calcineurin pathway, and the two-component pathways [2], [20] that are found across the fungal kingdom. Most of these signaling pathways are involved in regulating temperature stress. However, those pathways are also present in nonpathogenic species: how temperature is translated into morphogenesis in the pathogens remains an open question and the responsible thermosensors elusive. One promising candidate thermosensor is the histidine kinase Drk1, which triggers the temperature-dependent dimorphic transition in *Histoplasma* and *Blastomyces* [2]. Interestingly, its bacterial homolog DesK is the first documented histidine kinase that senses temperature fluctuations by detecting changes in membrane thickness [21]. The heat-shock chaperone Hsp90 plays important roles in the temperature-regulated dimorphism in *Candida*, and regulators Ryp1-3 are critical for the temperature-induced filament-to-yeast switch of *Histoplasma* [5], [6], [22]. However, these molecules likely sense temperatures indirectly *via* some unfolded proteins or act downstream of thermosensors. Besides proteins, RNAs with temperature-sensitive structures could also act as thermosensors, as shown in some bacterial pathogens [23].

In addition to exogenous factors (e.g., temperature, osmolarity, serum, pH, and sugar), endogenously produced small molecules such as quorum-sensing molecules (QSMs) affect fungal morphogenesis. QSMs regulate biological processes in a population density–dependent manner. In *C. albicans*, farnesol inhibits yeast-to-filament conversion in correlation with the inoculum size [24]. Tyrosol, on the other hand, stimulates yeast-to-filament conversion [25]. Multiple QSMs (e.g., farnesol, tyrosol, and morphogenic autoregulatory substance) likely fine-tune the dimorphic transition in *C. albicans*. Inoculum size is shown to affect morphotype transition in other dimorphic fungi [25], implying that QS systems are widely used to synchronize fungal cellular differentiation. Given that “anti-quorum sensing” is being exploited to curb bacterial virulence and that QS quenchers are less likely to select for resistance compared to conventional antimicrobial drugs [26], investigation into the QS response systems in fungi could help design effective treatments against mycoses.

## Alterations in the Cell Surface Are Underpinned by Cellular Morphology

Changes in cell shape or size are a visual manifestation of alterations in cell-wall properties. Although important, the physical aspects of shape and size may not by themselves be the key factor controlling virulence. Rather, alterations in cell surface molecules during morphogenesis help fungi to adapt to host conditions and to avoid or defend against host immune attacks. In *B. dermatitidis*, *H. capsulatum*, and *P. brasiliensis*, filament-to-yeast transition is accompanied by the increased production of α-1,3-glucan [14], [27], which masks the immunostimulatory β-glucan [28]. In *C. albicans*, the pathogenic hyphal growth form does not expose β-glucan to trigger antimicrobial responses [29]. In *C. neoformans*, spores germinate into yeast cells with enlarged capsule under host-relevant conditions. The capsule is immune-suppressive and antiphagocytotic, and it conceals antigens that could be recognized by the host.

A repertoire of cell-surface adhesion proteins (adhesins) are highly regulated during morphotype switches: some adhesins are specifically expressed in the pathogenic form, while others in the saprotrophic/commensal form. In *M. oryzae*, the integral membrane protein Pls1 regulates adhesion with Teflon-binding affinity to the plant surface and is specifically expressed in the appressorium [30], a cell type used for penetrating the plant tissue. In the human pathogenic fungi, adhesin Bad1 of *B. dermatitidis* is specifically expressed in the pathogenic yeast form [1], and it controls multiple processes during infection [31]. Adhesin Hwp1 in *C. albicans* is specifically expressed in hyphae to assist fungal attachment to epithelial cells, and Hwp1 is required for systemic candidiasis [32]. Als3, another adhesin expressed in *Candida* hyphae, binds to multiple host receptors and induces its own endocytosis to facilitate fungal penetration of epithelial cells [33]. By contrast, the adhesin Cfl1 in *C. neoformans* is associated with nonpathogenic filaments, and forced expression of Cfl1 in the pathogenic yeast form attenuates virulence [8]. Cfl1 is a downstream target of Znf2, which controls the expression of multiple adhesion genes in *Cryptococcus* (unpublished results). Accordingly, the *znf2*Δ mutant is more virulent than the wild type, even though both strains are in the yeast form during infection [8], [9]. Taken together, alterations in cell surface in addition to the physical cell shape and size likely underpin the link between morphogenesis and fungal virulence.

**Figure 2:**
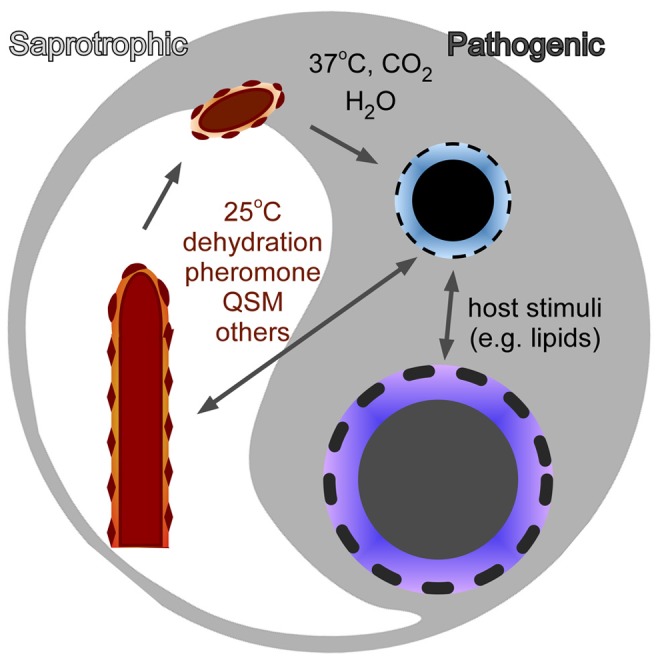
The transition between morphotype and virulence in fungi. Each morphotype has a unique cell-surface structure and composition. The differences in cell surface reflect differences in fungal cell physiology and contribute to the differences in the host immune responses elicited by these cells.

In the pathogenic fungi that are acquired from environmental sources, the filamentous form is generally not pathogenic. Nonetheless, adhesins or other molecules on filaments or spores help shape the initial interactions between the host and the pathogen (Figure 2), which establishes subsequent host-pathogen interactions. Success by these fungi to colonize the host, establish infections, and disseminate systemically is a combination of the downregulation of filament- or spore-specific molecules and upregulation of the yeast-specific ones. Therefore, investigation of cell-surface molecules specific to each morphological form will provide a new opportunity to comprehend host-fungus interactions.
